# Recent updates on correlation between reactive oxygen species and synbiotics for effective management of ulcerative colitis

**DOI:** 10.3389/fnut.2023.1126579

**Published:** 2023-07-20

**Authors:** Sumel Ashique, Neeraj Mishra, Ashish Garg, Belay Zeleke Sibuh, Pankaj Taneja, Gopal Rai, Sinouvassane Djearamane, Ling Shing Wong, Noura Al-Dayan, Shatabhisha Roychoudhury, Kavindra Kumar Kesari, Petr Slama, Shubhadeep Roychoudhury, Piyush Kumar Gupta

**Affiliations:** ^1^Department of Pharmaceutics, Pandaveswar School of Pharmacy, Pandaveswar, West Bengal, India; ^2^Amity Institute of Pharmacy, Amity University Madhya Pradesh, Gwalior, India; ^3^Department of P.G. Studies and Research in Chemistry and Pharmacy, Rani Durgavati University, Jabalpur, India; ^4^Department of Biotechnology, Sharda School of Engineering and Technology, Sharda University, Greater Noida, India; ^5^Department of Pharmaceutics, Guru Ramdas Institute of Science and Technology, Jabalpur, India; ^6^Department of Biomedical Science, Faculty of Science, Universiti Tunku Abdul Rahman, Kampar, Malaysia; ^7^Faculty of Health and Life Sciences, INTI International University, Nilai, Malaysia; ^8^Department of Medical Lab Sciences, Prince Sattam Bin Abdulaziz University, Al-Kharj, Saudi Arabia; ^9^Health Centre, Assam University, Silchar, India; ^10^Department of Applied Physics, Aalto University, Espoo, Finland; ^11^Laboratory of Animal Immunology and Biotechnology, Department of Animal Morphology, Physiology and Genetics, Faculty of AgriSciences, Mendel University in Brno, Brno, Czechia; ^12^Department of Life Science and Bioinformatics, Assam University, Silchar, India; ^13^Department of Life Sciences, Sharda School of Basic Sciences and Research, Sharda University, Greater Noida, India; ^14^Department of Biotechnology, Graphic Era Deemed to be University, Dehradun, India

**Keywords:** ulcerative colitis, synbiotics, gut microbiota, inflammation, oxidative stress, superoxide dismutase

## Abstract

Ulcerative colitis (UC) is presently considered a multifactorial pathology, which may lead to persistent inflammatory action of the gastrointestinal tract (GIT) because of an improperly managed immunological reactivity to the intestinal microbiota found in the GIT. The immune response to common commensal microbes plays an essential role in intestinal inflammation related to UC synbiotics, and it is an important element in the optimal therapy of UC. Therefore, synbiotics, i.e., a mixture of prebiotics and probiotics, may help control the diseased state. Synbiotics alleviate the inflammation of the colon by lowering the reactive oxygen species (ROS) and improving the level of antioxidant enzymes such as catalase (CAT), glutathione peroxidase (GPX), and superoxide dismutase (SOD). Prebiotic supplementation is not a common practice at the moment, despite numerous research findings proving that the benefits of both probiotics and prebiotics encourage their continued existence and positioning in the GIT, with positive effects on human health by managing the inflammatory response. However, the fact that there have been fewer studies on the treatment of UC with different probiotics coupled with selected prebiotics, i.e., synbiotics, and the outcomes of these studies have been very favorable. This evidence-based study explores the possible role of ROS, SOD, and synbiotics in managing the UC. The proposed review also focuses on the role of alteration of gut microbiota, antioxidant defense in the gastrointestinal tract, and the management of UC. Thus, the current article emphasizes oxidative stress signaling in the GI tract, oxidative stress-based pathomechanisms in UC patients, and UC therapies inhibiting oxidative stress’ effects.

## 1. Introduction

Ulcerative colitis (UC) is a gastrointestinal inflammatory condition characterized by bloody and mucous diarrhea, rectal bleeding, and gastrointestinal pain (1). According to current projections, UC affects around 5 million people worldwide and is characterized by recurring and repatriating irritation of the intestine’s mucous membrane. In UC, inflammation sticks to the mucus layer (2, 3).

Ulcerative colitis is associated with impaired mucosal barrier function, which allows luminal bacteria to generate a prolonged and uncontrollable inflammatory response. UC is a type of sickness classified as one of the “inflammatory bowel diseases (IBDs).” It is defined by a prolonged inflammatory response of the “intestinal lamina propria” that might start in the rectum and progress across the colonic mucosa. Clinically, UC and Crohn’s diseases frequently contribute significantly to global mortality, particularly in the Western world (4).

Unlike Crohn’s disease, where the mucosa surrounding the ulcers may or may not be inflamed, ulcers in UC are virtually invariably accompanied by mucosal inflammation (5). Interleukins (ILs), important components of the cytokine profile observed in the gastrointestinal mucosa in UC, have therefore been emphasized as potential targets for targeted therapeutics in the future. The ILs chosen for consideration have the highest promise as future targeted treatments. Furthermore, investigating several of the most recently investigated ILs involves their potential significance in UC (6). In addition to the standard proinflammatory cytokines like IL-1, IL-6, and TNF-, a complex network of Th2 cytokines, including IL-10 and IL-13, play an important role in the pathogenesis of UC. This network is crucial because it regulates the immune response.

In comparison to Crohn’s disease, UC affects a smaller geographic region. The sickness only invades (inflames) the inner lining of the gut tissue, and it usually only affects the colon (large intestine), including the rectum and anus. The prevalence of UC is far greater than that of Crohn’s disease. North America and northern Europe have the highest UC prevalence and incidence rates (7). The incidence rates vary between nine and twenty cases per 100,000 people per year, whereas the prevalence rates range between 156 and 291 instances per 100,000 people. The etiology and pathophysiology of UC are both complex. The cause of UC is assumed to be an imbalance between the intestinal microbiota and mucosal immunology, which causes excessive inflammation in the digestive system (8). As a result, an imbalance in the digestive tract microbiota has a role in the pathogenesis of UC. The intestinal microbial population and gut bacteria’s functional diversity and stability are all affected in individuals with UC. Certain Firmicutes bacteria are declining, while Bacteroidetes bacteria and facultative anaerobes are increasing (9). Dysbiosis has been observed in UC patients (10), albeit to a lesser extent than in Crohn’s disease patients. Patients with UC have been reported to have lower biodiversity (11), with fewer Firmicutes and a higher proportion of Gamma-proteobacteria and *Enterobacteriaceae* in their gut microbiomes. Furthermore, individuals with the illness have more sulfite-reducing Delta-proteobacteria in their colons (12).

Probiotics are living microorganisms with a wide range of beneficial features which play an important role in GIT protection (13). However, probiotics have a wide range of impacts on the human body, including the skin, oral cavity, respiratory tract, urinary tract, and reproductive tract. Clinical trials have investigated probiotics’ health advantages in children, adults, the elderly, and immunocompromised patients (14). Probiotics work through several methods, including gut flora alteration, intestinal mucosa barrier strengthening, pathogen colonization decrease, inhibition of enhanced immune response, and generation of short-chain fatty acids, amino acids, vitamins, and enzymes, among others. The use of probiotics as part of a UC treatment approach is becoming more widespread. Probiotics are living microorganisms that do not cause sickness. Lactobacillus, Bifidobacterium, and Enterococcus are examples of probiotic bacteria. Probiotic bacteria have been found in studies to be beneficial, particularly to the gut and immune systems (15). Probiotics have been proven to boost both local and systemic immunity, repair the function of a disrupted mucosal barrier, correct an imbalance in the intestinal microbiota, reduce competition between potential pathogens, and encourage intestinal barrier function (15, 16). Probiotics can effectively induce and prolong remission in UC patients, indicating that UC care should emphasize favorable gut flora (17–21).

On the other hand, a synbiotic is a mix of both a probiotic and a prebiotic, a carbohydrate that functions as a food source for the probiotic and allows it to develop more efficiently in the gut. As a result, a synbiotic does not include living microbes. Synbiotics include both probiotics and prebiotics; this combination is thought to be more helpful in gut health and function than either probiotics or prebiotics alone (22). As a result, considering the numerous possible combinations, the use of synbiotics, in which probiotics and prebiotics act together to give a synergistic effect, is considered promising (22, 23).

The purpose of synbiotic involvement, on the other hand, is uncertain. More research is needed to determine the synbiosis in both prebiotics and probiotics that could modify inflammatory reactivity primarily through inflammatory cytokines and innate immune activation, as well as the development of reactive oxygen species (ROS)-inhibiting short-chain fatty acids (SCFAs) associated with intestinal mucosal stabilization, T cell initiation, advancement of anti-inflammatory cytokines efflux, and inflammation reduction (3).

## 2. Pathogenesis of ulcerative colitis

Ulcerative colitis is a “chronic IBD” that affects the rectum and the colon. Several factors, including genetic profile, environmental and gastrointestinal conditions, and mucosal immune dysfunction, are thought to impact the genesis of UC. Despite its broad prevalence, the pathophysiology of UC is complicated and poorly understood. Nonetheless, the newly accessible data allow for constructing a current working model of the disease’s pathophysiology. This model incorporates several aspects and components that contribute to illness development. UC is an intestinal barrier disorder caused by a breakdown in an epithelial cell or the fundamental epithelial architecture of the gastrointestinal tract. UC can be caused by various factors, which might eventually lead to immunological difficulties.

Furthermore, infected individuals may risk getting a disease caused by commensal gut microorganisms (24). Alternatively, the barriers might be disrupted by highly inflammatory chemicals and cells in the lamina propria, leading to the barrier’s rupture; this inflammatory cascade would then contribute to the illness’s chronicity. The pathogenesis of UC is complex, with several factors. SCFAs are generated by probiotic gut bacteria from a fiber-rich diet that cannot be taken directly (25). SCFAs, including acetate, propionate, and butyrate, are essential metabolites for sustaining intestinal homeostasis (26). SCFAs with significant anti-inflammatory action can reduce ROS production, which may modulate the immunological function and prevent an excessive immune response, delaying the clinical development of IBD. SCFAs are essential for fueling intestinal epithelial cells and are known to maintain gut barrier function. SCFAs contribute to the formation and development of UC (25–27). In UC, the mucous membrane fails to generate as much intestinal mucin. A barrier breach is produced by changed microbiota and a weaker mucous membrane, which allows the microbiota to permeate the epithelial barrier more easily. Apoptotic foci and altered tight junction protein expression damage the intestinal epithelium, enabling germs to get through, activating macrophages and antigen-presenting cells (APCs), and promoting the production of chemokines that attract neutrophils. Neutrophil extracellular traps act as the first line of defense, and immune cells infiltrate by sticking to blood vessel endothelial adhesion molecules. Type 1 T-helper (TH1) cells become polarized as a result of invading monocytes that grow into macrophages producing tumor necrosis factor (TNF), interleukin (IL)-12, IL-23, and IL-6.

Furthermore, IL-36 generated by the epithelium inhibits regulatory-T (Treg) cells, causing IL-9-producing T-helper (TH9) cell polarization. IL-13, released by natural killer (NK) T cells, also causes barrier dysfunction. Changes in barrier function in UC patients can involve cytolytic destruction to the epithelial layer by NK-T cells and a more subtle modification caused by the actions of IL-13. Consequently, this cytokine may have a dual pathogenic effect, one influencing epithelial cells directly and the other as a stimulator of NK T cell cytotoxicity (28).

## 3. Alteration of gut microbiota in ulcerative colitis

Numerous investigations have shown that UC patients have impaired gut microbiota regarding composition and structure. There is a reduction in several bacteria, including *Akkermansia muciniphila*. It is a common element of the mammalian gut microbiota, accounting for 1 and 5% of all human intestinal microorganisms. According to the observations of numerous studies, there is a connection between UC and *A. muciniphila*. Together with the Roseburia bacteria, the *A. muciniphila* levels were found diminished in patients with UC (29). Patients with UC typically notice changes in their gut microbiota composition. Furthermore, several investigations suggest that abnormalities of the gut microbiota are strongly associated with UC (30). The gut microbiota significantly influences the gut mucosal immune system (21, 31–33).

## 4. Oxidative stress signaling in the gastrointestinal tract

**Figure 1:**
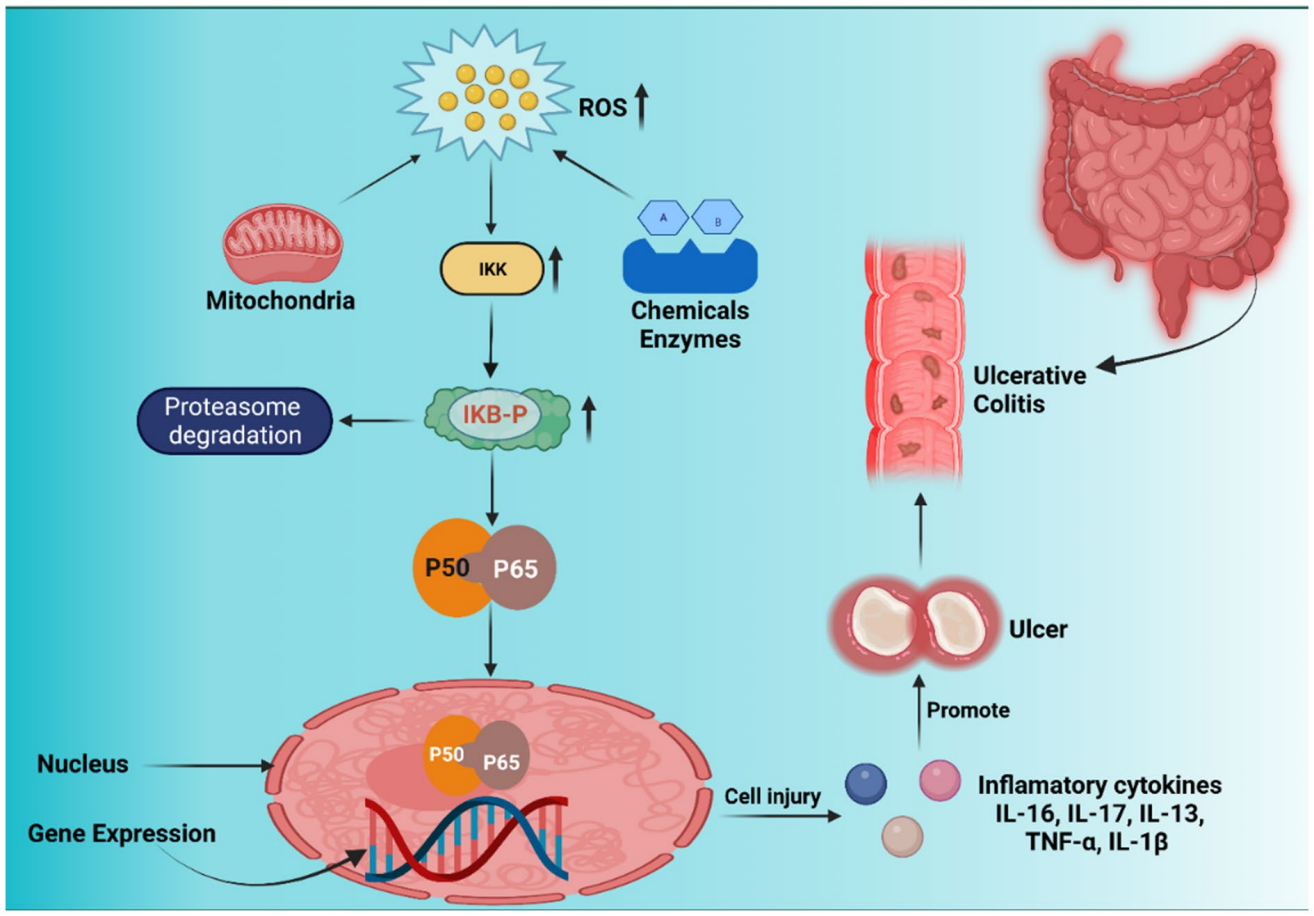
Nuclear factor kappa-B (NF-κB) signaling pathway, inflammation, and ROS-induced carcinogenesis. Inhibitor of nuclear factor-κB (IκB) kinase (IKK) is first activated by xenobiotics, excessive reactive oxygen species (ROS) from the mitochondrial membrane, and enzyme reactions. IκB is phosphorylated by activated IKK, which causes IκB (inhibitor of nuclear factor kappa-B) to be ubiquitinated and broken down by the proteasome, releasing NF-κB proteins (p50 and p65). Free p50 and p65 shift target gene expression in nuclei identical to inflammatory cytokines, resulting in inflammatory lesions and carcinogenesis.

ROS are necessary for mammalian cell survival. Free radicals such as hypochlorous acid (HOCl), hydrogen peroxide (H_2_O_2_), peroxyl (RO_2_), hydroxyl radicals (H.O.), superoxide (O_2_^−^), and others are referred to as ROS (34). ROS and RNS are two important components in the cell that cause harm to nucleic acids, lipids, proteins, and carbohydrates and regulate the gene transcription that triggers immunological activities in the GIT (35). Intrinsic ROS is mostly produced in cell constituents such as the cytosol, nucleus, peroxisomes, mitochondria, and endoplasmic reticulum. The electron transport chain (ETC) primarily contributes to ROS production. Also, intracellular ROS is produced by a variety of enzymes, including cyclooxygenases (COXs), nitric oxide synthase (NOS), myeloperoxidase (MPO), glucose oxidase, xanthine oxidase (XO), NADPH oxidase (NOX), and peroxidases. Various cytokines produced by Th2-type T cells, such as IL-13, IL-10, IL-5, and IL-4, help to suppress UC. Numerous opposing effects maintain GI epithelial stability while destroying UC and gastric ulceration (36). Xenobiotics, medications, alcohol consumption, antigens (luminal), smoking, chemotherapy, and radiation are some of the external variables that cause ROS generation in UC. Due to its antioxidant capabilities, the cells can tolerate a certain quantity of ROS under ordinary circumstances, which is critical for GI equilibrium. However, an excessive oxidant payload promotes increased ROS production, irritation, transmembrane permeability, DNA destruction, and eventually UC (34–37) (Figure 1).

## 5. Antioxidant defense in the gastrointestinal tract

Uncontrolled oxidative stress harms the GIT and the human body’s antioxidative defensive mechanism (s) that could protect individuals from the consequences of too much ROS. The defense mechanism indicates that the number of ROS the human body produces can be managed without causing damage. Numerous enzymatic antioxidants, including CAT, GPX, and SODs, are found in the exogenous antioxidant defense systems. Multiple diagnostic outcomes have previously demonstrated that IBD sufferers have three SOD isoforms, with SOD2 being significantly highly expressed, SOD1 being less impacted, and SOD3 being decreased mostly in intestinal epithelial cells (IECs). SOD performance is usually associated with the degree of UC in IBD patients, wherein S.O.D. activity is increased during IBD pathophysiology and promotes oxidative injury (35).

## 6. Pathomechanisms of oxidative stress in ulcerative colitis patients: role of gut microbiota

Though several factors are responsible for UC, such as genetic perceptivity, alterations in IECs, immune dysregulation, microbiota resistance, and ecological variables – all contribute to the progression of IBD. However, our major focus is the correlation between ROS and UC.

GIT is vulnerable to ROS assault. However, it is exposed to the external ecosystem, which includes the immune system, intestinal flora, and dietary components, all of which are significant sources of ROS. The GIT generates ROS primarily via two biochemical responses: the NADPH oxidase mechanism and the HX/XO pathway. Elevated levels of ROS can disrupt cell components (38, 39), especially structural proteins, and eventually increase gut permeability and disrupt the GIT membrane, resulting in GIT irritation. The microflora is linked to various defensive, architectural, and biochemical functions and is essential to gut equilibrium and recipient health. Gut bacteria regulate the development of pathogenic bacteria in the GI system, activate immunity, regulate vitamin and mineral utilization and host metabolic activity, produce SCFAs, disrupt carbohydrates and proteins important for mucous membrane & cell function, and the production of anti-inflammatory IL (40). Recent research has shown that the equilibrium between the intestinal microbiome in the GIT and human defense mechanisms is critical in the etiology and persistence of UC (41). Dysbiosis (alterations in the makeup of commensal microorganisms or instability y in the microbiota environment) has been documented in UC individuals, and current therapies also impact the microbiome (42). As a result, an investigation into establishing synbiotic-associated therapeutics for UC is currently proceeding. In systemic inflammation, intestinal microbes activate the formation of NO and NOS via host macrophage stimulation, initiating DNA damage. Oxidative stress causes DNA damage, protein deposition, and cell wall disorganization and activates the beginning of inflammatory reactivity via supportive remarks, triggering supplementary ROS development and further membrane/tissue injury (43).

## 7. Targeting oxidative stress in ulcerative colitis

Proinflammatory cytokines like NOS and ROS are more involved in the genesis and progression of UC (44). In the acute inflammatory lamina propria of individuals affected by UC connected to the endothelium, a noteworthy infiltration of white blood cells and improved myeloperoxidase (MPO) concentration were assessed (45). In UC, iNOS is the primary key element responsible for increased NO synthesis in the epithelial cell and iNOS-derived NO promotes TNF-α-expansions in the intermediate and proximal intestine, which increases leukocyte infiltration primarily through activation of the formation of “intracellular adhesion molecule” (ICAM) and P-selectin, resulting in intestinal cellular injury (46). The influx of neutrophils and the activation of important genomic signal transduction pathways, including “AP-1” and “nuclear factor-kappa B (NF-kB),” increase inflammatory activity and tissue destruction (47).

## 8. Therapies inhibiting oxidative stress in ulcerative colitis

IBD is a severe gastrointestinal condition characterized by immunological dysregulations, supporting the hypothesis that IBD therapies should primarily focus on reducing inflammation. In addition, anti-inflammatory medicines such as corticosteroids, infliximab, mesalazine, and sulfasalazine are used in conventional therapeutic approaches to combat irritation promptly and alleviate IBD discomfort. These bio-actives work by inhibiting NF-κB-or TNF-α-associated inflammation, resulting in various health consequences such as GIT issues, anemia, allergy, and medication resistance. Another category of therapeutics, immune modulators like thiopurines and cyclosporine, can also be used for managing IBD via immunosuppression (48). These immune modulators are frequently associated with anti-inflammatory chemicals. Most have free radical-scavenging abilities, with some resulting from TNF-α-induced downstream antioxidant effects. Because of the significant growth in the complexity of UC and colon cancer, there is a greater need for innovative treatment methods for UC. Various experimental studies have identified one of the key oxidative stress pathways in IBD, and modulating Nrf2 signaling and blocking ROS development by inhibiting mitochondria and NOX are both critical therapy options for IBD. As a result, several promising alternative treatment modalities with antioxidant properties, such as ROS blockers, responsive nutritional approaches, and naturally derived agents that prevent apoptosis and activate antioxidant activity, have received considerable focus as “complementary and alternative medicines” (CAMs) in the management of UC (49–56) (Table 1).

**Table 1:** Antioxidative and anti-inflammatory effects of therapeutics used to manage ulcerative colitis (UC).

Agents used in UC management	Outcomes	References
N-acetylcysteine	Decreased lipid peroxidation, enhanced GSH and SOD in ulcerative colitis, and decreased iNOS activity in UC	(49)
Tetradecylthioacetic acid	Reduced iNOS, TNF-α, and IL-6 mRNA in ulcerative colitis UC	(50)
Mesalazine	Decreased O_2_•^−^, H_2_O_2_ in UC, ↓IL-6, Il-8, reduced GSH, TNF-α in UC	(51)
Glucocorticoids	Reduced MPO and neutrophil elastase in pediatrics IBD	(52)
Infliximab	Reduced TNF-α in the colonic mucosa, reduced INF-γ mRNA in inflammatory cells in colitis	(53, 54)
Tributyrin	Enhanced TGF-β and IL-10 in lamina propria	(55, 56)

## 9. Role of probiotics for the management of ulcerative colitis

Probiotics have beneficial effects on the host microbiota and have attracted greater research interest in managing UC. Probiotics serve as an inhibitor of ROS production, the development of antioxidative enzymes, metal chelation, and enzyme suppression. Probiotics have increased GPX, CAT, SOD, and GSH levels while reducing NO and MPO functions (57). The multifaceted activity of nutrition, nutritious dietary compounds, and healthy gut microbiome has bolstered the use of probiotics, which have been shown to have favorable effects on the human intestinal microbiota (58). Many microbiomes have been studied for discarding gut microbiota. Microbes such as (*Streptococcus*, *Bifidobacterium*, and *Lactobacillus*) were used in the synthesis of probiotic strains associated with more positive treatment responses on GIT inflammation and the ability to maintain a better and healthier intestinal microbiota, and probiotics are often used to assess the efficacy of living microorganisms in reducing IBD manifestations (59). Scientific studies using probiotics to treat IBD are widespread (60). As a result, whenever the gut ecosystem has been damaged by illness, ill-nutrition, or drugs, better knowledge is required when selecting a particular probiotic strain that might alter the patient’s health. Probiotics have been offered as a novel preventative and therapeutic alternative in cancer management and may inhibit cancer development. Thus, probiotics could provide a fresh approach to studying the active ingredients found in various probiotic strains. With few traditional therapies available, there is a need for new options. One such strategy is the delivery of chemotherapeutic drugs via nanocarriers employing nanotechnology (61).

Mesalamine and probiotic “(*Saccharomyces boulardii* and *Lactobacillus acidophilus*)” encapsulated pectin microparticles embedded with cellulose acetate phthalate (CAP) were synthesized by Singh et al. in 2021. The main issue with this research is the strong NO scavenging capacity of *Saccharomyces boulardii*, which was validated by the NO test. According to the FT-IR interpretation, no chemical interaction between the medication and CAP was seen. According to the *in vitro* drug release kinetics of coated microparticles, the synthesized formulation can release the medicine and probiotics at the colonic site (62). To control UC, Singh et al. (63) have developed and characterized “enteric-coated pectin pellets” consisting of mesalamine and *S. boulardii* for precise colon-targeted drug delivery. Mesalamine and *S. boulardii* pellets were created utilizing the extrusion spheronization method, pectin, and microcrystalline cellulose (MCC) and were then decorated with cellulose acetate phthalate (CAP). Experimental studies have demonstrated that mesalamine and *S. boulardii*-coated pellets dramatically alleviated the sick conditions in Wistar rats (63).

### 9.1. Mechanism of probiotics against ulcerative colitis

**Figure 2:**
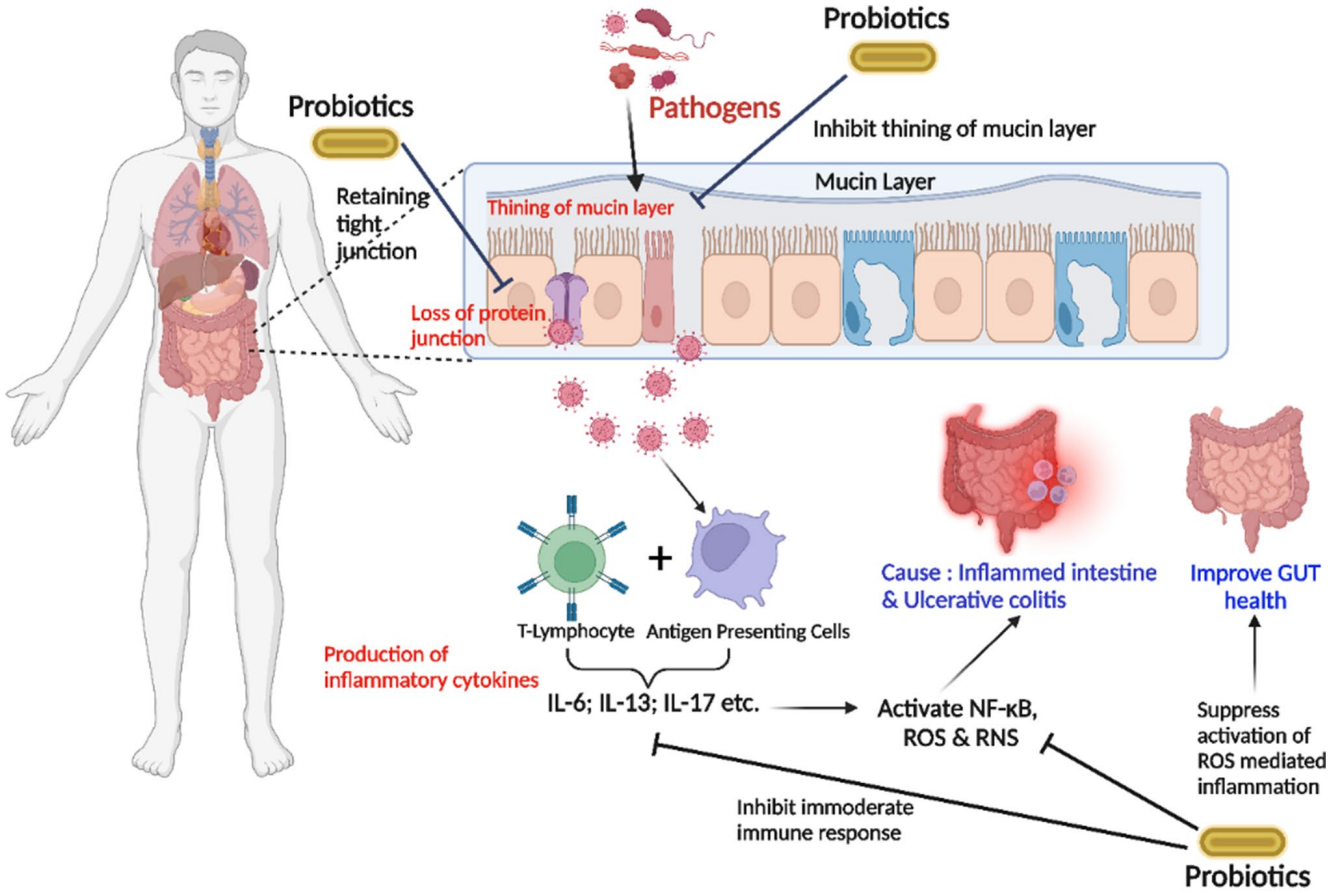
The strategy of probiotics’ action on the gut lumen is associated with inhibiting the inflammation in ulcerative colitis (UC). The entry of an overwhelming number of pathogenic microorganisms, the degradation of cell-junction proteins, and the thin mucin layer trigger inflammatory reactions. T-lymphocytes produce proinflammatory cytokines after pathogens are identified by “antigen-presenting cells (APC),” stimulating inflammation-inducing NF-κB and producing reactive oxygen species (ROS) and reactive nitrogen species (RNS), resulting in an acute inflammatory intestinal lumen. Probiotics inhibit excessive immunological responses and attenuate ROS-induced inflammatory responses (Interleukin-6; Interleukin-13; Interleukin-17).

Abundant pathogenic microorganisms, depletion of protein bindings and junctions, and a thinner mucus membrane cause inflammatory consequences in UC. Although the APCs identify microorganisms, T-lymphocytes form proinflammatory cytokines that trigger inflammatory mediators NF-κB, which produce reactive nitrogen species (RNS) and ROS, resulting in the irritated intestinal mucosa. Being overweight causes abnormalities between microorganisms and commensal bacteria and a significant irritation effect. An increased ω-3 triggers inflammatory reactions to ω-6 fatty acid equilibrium in the diet. The use of probiotics aids in the maintenance of functional gut flora by preserving barrier function and the mucus barrier. Probiotics and antioxidants decrease irrational immune function and ROS-associated inflammatory responses modified by antioxidants (64) (Figure 2).

## 10. Selective therapies aiming at microbiota manipulation in ulcerative colitis

Probiotics, prebiotics, antibiotics, gut microbiome transplants, and a nutritious diet may all assist in maintaining a balanced gut microbiota habitat. Antibiotics are excellent in eliminating pathobionts, but their non-selective antibacterial activity disrupts gut equilibrium by destroying beneficial microflora, reducing their usage in managing colorectal cancer.

### 10.1. Studies on the use of prebiotics and probiotics in the management of ulcerative colitis

Host bacteria utilize nutritional prebiotics to give therapeutic advantages to target tissues. Prebiotics are thought to increase intestinal irritation by promoting beneficial gut microbiota formation, intestinal vulnerability, and SCFA production. Probiotics are live microorganisms that can provide therapeutic effects to humans whenever administered at sufficient levels. They are usually made up of one or even more strains of bacteria (65–72) (Table 2).

**Table 2:** Several findings of probiotics and prebiotics against ulcerative colitis (UC).

Prebiotics and probiotics	Outcomes against UC	References
Lactulose	Reduced inflammation	(65)
Oligofructose enriched Inulin	Levels of fecal calprotectin increased in the prebiotic-administered group than in the placebo-taken group	(66, 67)
UC treated with different doses of oligofructose-enriched inulin	Prebiotic courses were found to be higher butyrate levels	(68)
Meta-analysis of *Escherichia coli* Nissle 1917, *Bifidobacterium longum* 356, *Lactobacillus rhamnosus G.G*., a multi-strain probiotic containing a combination of lactic acid bacteria, *Streptococci*, and *Bifidobacterium* probiotics	The use of probiotics reduced adverse events	(69)
Meta-analysis of randomized controlled trials (RCTs) examining the effects of probiotics, prebiotics, and synbiotics on human UC	Patients suffering from active UC who took *Bifidobacterium*-containing probiotics were more likely to be in remission than the placebo group	(70)
Supplementation of *Bifidobacterium*-fermented milk	Exhibited anti-inflammatory properties by protecting mucosal barrier integrity and maintaining gut microbiota homeostasis	(71, 72)

## 11. Cross-links between reactive oxygen species and microflora in the gastrointestinal tract

ROS, which comprises radical variants (superoxide) and non-radical peroxide forms (H_2_O_2_), are short-lived, highly electrophilic entities that originate from the partial reduction of molecular oxygen. Extremely reactive ROS, particularly superoxide, may cause macromolecular destruction to crucial biological constituents, including membrane phospholipids and nucleic acids. Another important finding from scientific studies was the evidence that certain species of commensal gut bacteria in humans cause the quick, “deliberate” production of physiological amounts of ROS in human epithelial cells (35). Additionally, epithelial cells co-cultured with specific bacteria demonstrated an increment in the oxidation of soluble redox sinks like glutathione and thioredoxin, as well as an enhancement in redox-stimulated transcriptional stimulation, both of which reflect a cellular response to elevated ROS. Contacting cells may produce significantly varying ROS levels in response to commensal bacterial strains. Although all examined microorganisms have some capacity to change the intracellular redox state, *Lactobacilli* are potent inducers of ROS production in cultivated cells. High ROS-stimulating microorganisms, like *Lactobacilli*, may have membrane elements or even release substances that stimulate cellular ROS generation. Bacteria that produce high levels of ROS may have improved adhesion or the capacity to permeate mucous membranes, providing them with more proximal accessibility to cellular receptors (e.g., FPRs and TLRs). FPRs are potential candidates and are reported to substantially induce ROS generation, located on apical surfaces and in epithelial cells and phagocytes. The gut has a variety of unique cell varieties that transmit proinflammatory and immune-tolerance messages in response to commensal bacteria and pathogens. The generation of ROS by mucosa-resident cells or freshly recruited innate immune cells is crucial for antimicrobial responses and the control of signal transduction pathways, including phenomena associated with wound healing. Crohn’s disease and pancolitis have been linked to decreased ROS production due to patient variations that render NADPH oxidases as passive sources of ROS. However, ileitis and UC have been related to increased ROS production due to upregulated oxidases or modified mitochondrial features and functions (35, 73).

Its pathogenesis is multifactorial, including environmental factors, genetic susceptibility, epithelial barrier defect, symbiotic flora imbalance, and dysregulated immune response. Thus far, although immune cells have become the focus of most research, it is increasingly clear that intestinal epithelial cells play an important role in the pathogenesis and progression of UC. Notably, apoptosis is a vital catabolic process in cells, which is crucial to maintain the intestinal environment’s stability and regulation of intestinal ecology (74). ROS have been recognized as a common mechanism in UC (75). Either antioxidants or free radical scavengers are reported as effective therapeutic agents for UC (76, 77).

Moreover, due to the relatively high ROS concentration in UC patients’ tissues, ROS-responsive systems may specifically release drugs in inflamed colon tissues. Long-term irreversible damage to the GI structure and function in patients with IBD increases the risk of colon cancer. Current treatment strategies include corticosteroids, aminosalicylic acid (ASA), immunomodulatory drugs, Janus kinase inhibitors, and biological agents-monoclonal antibodies against TNF-α, IL-12/23 (78).

## 12. Role of microbiome, synbiotics, and xylooligosaccharides in the management of ulcerative colitis

Several studies have revealed that UC is closely linked with disturbance in gut microbiota (79). Gut microbiota plays a main role in a healthy gut mucosal immune system (80). Scaldaferri et al. (81) established that the most severe inflammatory sites in the gut of UC patients are also the sites with the highest abundance of bacteria. When the dominant bacterial species in the gut is altered, this results in instability of the gut microbiota and an immune reaction within the gut mucosa (82). Microbial disorders can cause deviations in the metabolism of bacteria, inducing gut inflammation. Variations to innate gut microbiota characteristics may be used as a diagnostic marker and a prognosticator of UC (83).

Numerous strains of probiotics or prebiotics, in varying ratios, may be used to sustain healthy microbiota. Synbiotics are a mixture of prebiotics and probiotics that are more effective than individual prebiotics and probiotics. Synbiotics synergistically impact the intestinal microbiota, enhancing certain advantageous probiotic strains’ durability and physiological functions. Bifidobacteria, Lactobacilli, inulin, oligosaccharides, and fibers as prebiotic elements are most often utilized in synbiotic combinations. Due to their stronger potential to increase SCFAs developing bacteria counts and substrates for fermentation, the synbiotics get a more substantial anti-inflammatory impact, either probiotics or prebiotics individually (22, 23, 25).

UC is intimately linked to gut microbial dysbiosis. Prebiotic treatment is a viable strategy for managing UC, particularly sustaining remission. “Xylo-oligosaccharide (XOS)” is an effective prebiotic with several clinically documented medical advantages and few adverse outcomes. Prebiotic Xylo-oligosaccharide (XOS), which improves gut flora, is more effective than conventional prebiotics (84). The term “prebiotic” describes non-viable dietary components like “fructan (also known as “inulin”) indigestible polysaccharides, galactooligosaccharides (GOS), oligosaccharides, or fructooligosaccharides (FOS), Xylo-oligosaccharides (XOS)” that preferentially promote the growth of a small number of health-promoting microorganisms in the gut and have beneficial impacts on the GIT, cognitive abilities, cardiovascular wellness, and bone density (85, 86). To explain the potency of prebiotics and Xylo-oligosaccharides, numerous research works were carried out to show the beneficial effect of prebiotics and xylo-oligosaccharides in the maintenance of gut and UC.

A synbiotic is a good option for reducing UC-related inflammation since it contains both probiotic and prebiotic components. To ascertain the additive effect of the probiotic “*Bifidobacterium infantis* (*B. infantis*)” and the prebiotic “xylooligosaccharide (XOS)” against ulcerative colitis, Sheng et al. (87), conducted a study on the synbiotic supplementation containing “*Bifidobacterium infantis* and xylooligosaccharides.” For this, “*B. infantis*, XOS, or synbiotic (a mix of *B. infantis* and XOS)” were administered to “C57BL/6 mice” for 21 days. “Dextran sulfate sodium (DSS)” solution in water was given to the mice during the last 7 days of therapy to cause colitis. The “disease activity index (DAI)” and pathological scores were all reduced by all treatments, suggesting that synbiotic therapy was more effective than either probiotic or prebiotic treatment alone. All treatment groups significantly reduced the proinflammatory cytokines “TNF-α and IL-1β” compared to the DSS-induced colitis group. All treatments reduced oxidative stress, and the mRNA levels of the “tight junction (TJ) molecules zonula occludens-1 (ZO-1), occludin, and claudin-1” were elevated in the colon tissues. As a result, the reported effectiveness of synbiotics against colitis may be explained by the additive interaction of the probiotic and prebiotic components’ direct anti-inflammatory actions and their capacity to strengthen the integrity of the colonic epithelial barrier. According to research findings, synbiotics are a viable dietary supplement or functional food for the efficient treatment of UC (87). Another research used an *in vitro* fermentation model to examine the prebiotic impacts of XOS on the fecal microbiota of individuals with UC who were in clinical remission. The research included five UC patients in clinical remission and five healthy participants. Fresh feces specimens from UC patients were diluted and inoculated in “yeast extract, casitone and fatty acid (YCFA) medium,” either alone or in combination with XOS. Samples were obtained for “16S rDNA” sequencing to examine the makeup of the gut microbiota after 48 h of fermentation. Using original fecal samples, differences in the gut microbiota between healthy individuals and UC patients in clinical remission were found. The effects of XOS on the gut microbiota of UC patients were then shown by comparing the differences between the YCFA medium alone or with XOS samples. The fecal samples of UC patients were different from those of healthy volunteers in “principal coordinate analysis (PCoA) and principal component analysis (PCA).” The relative abundances of “g Roseburia and g-Lachnospiraceae ND3007 group” were greater in healthy volunteers than in UC patients, but “o-Lactobacillales” abundance exhibited the reverse tendency, according to a “linear discriminant analysis effect size (LEfSe) analysis.” The abundances of the “g-Eubacterium halli group” and “g-Lachnospiraceae ND3007 group” were greater in the healthy volunteers than in the UC patients (P 0.05) according to the Wilcoxon rank-sum test bar plot. The Wilcoxon rank-sum test also revealed that XOS fermentation in UC patients boosted the development of bacterial groups such as “g-Roseburia, g-Bifidobacterium, and g-Lactobacillus,” which is advantageous for the recovery of intestinal disorders. These findings point to XOS as a potential prebiotic material for sustaining clinical remission by alleviating dysbiosis in the feces of UC patients (88). Another study by Le et al. in 2022, compared the effects of soymilk inoculated with “*Lactobacillus rhamnosus* GG (LGG) and *Weissella cibaria* FB069 (FSMXW),” reported a synbiotic fermented soymilk fortified with XOS, on the growth of colon cancer cells. FB069 and LGG could expand in soy-based products, and fermentation quickly lowered their pH. In fermented soymilk inoculated with *W. cibaria* FB069, adding XOS dramatically increased the acidification rate, viscosity, and total cell concentration. However, after receiving the LGG vaccine, the same result was not seen. The synbiotic FSMXW also had increased “dextran, folate, GABA, and aglycone” levels. Lowering the transcription of “MD2, TLR4, MyD88, and NF-κb, FSMXW” reduced the growth of the Caco-2 and HCT-116 cell lines. The synbiotic soymilk containing XOS and *W. cibaria* FB069 through fermentation increases nutrients and useful compounds. The research outcome indicated that *W. cibaria* and XOS may be used to create functional foods and healthcare items (89). The data also imply that XOS can treat dysbiosis in individuals with UC who have achieved clinical treatment; hence, XOS may constitute a viable prebiotic for the therapy of UC.

**Figure 3:**
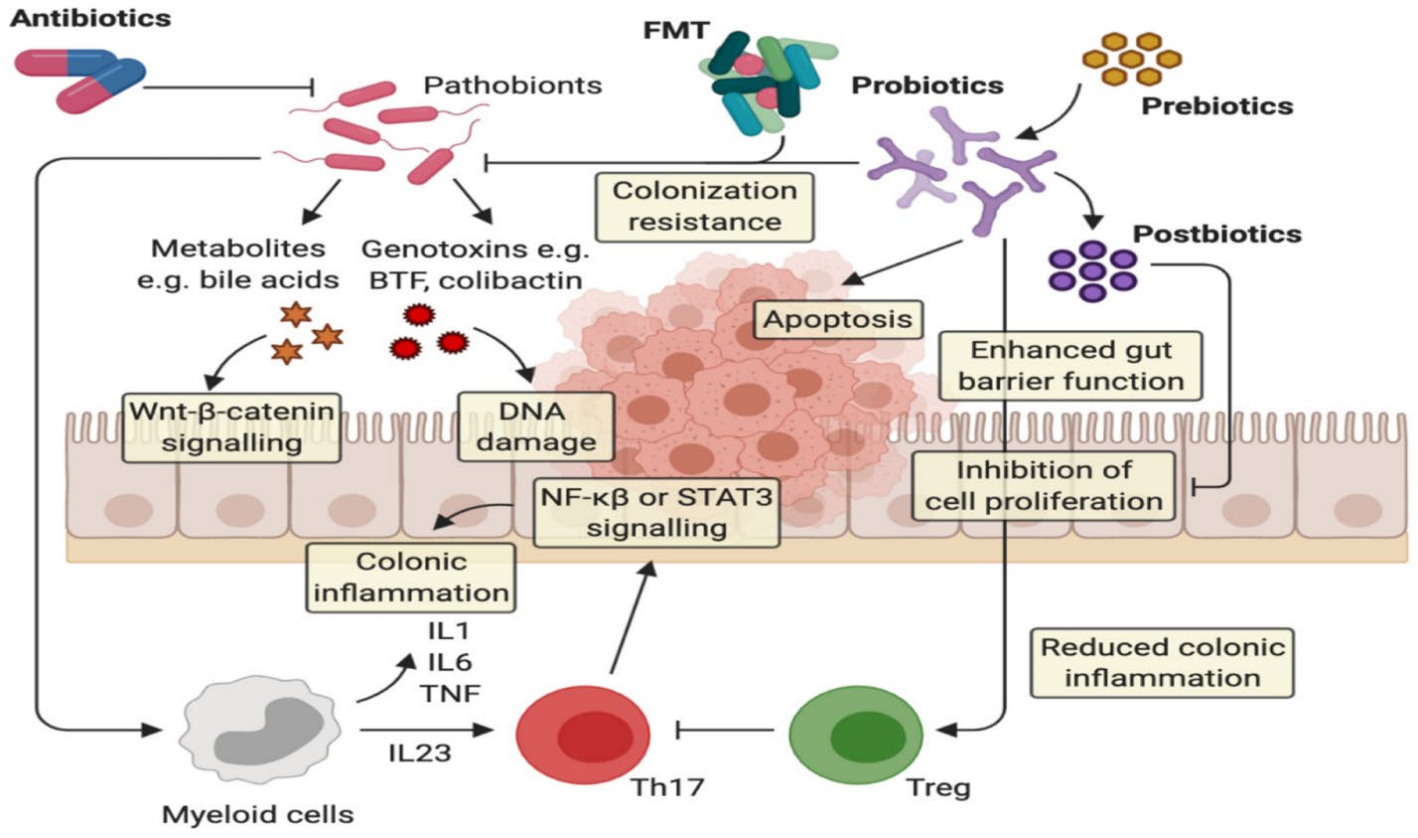
The implication of prebiotics and probiotics in ulcerative colitis. Probiotics act via several anti-cancerogenic mechanisms: (i) probiotics can inhibit the colonization of pathogenic bacteria, (ii) they can improve the barrier function by enhancing mucin production and tight junction protein appearance, and (iii) they Improve homeostatic immune responses, providing the extension of anti-inflammatory responses via Treg cells and the modification of proinflammatory cytokine release, (iv) Increase apoptosis on inflamed cells.

Symbiotic medication is a unique way to improve the operating performance of any immune-related illness, and further therapeutic, prospective studies are needed to confirm positive results in UC. Various animal investigations have lately been undertaken to assess the effectiveness and safety of synbiotics on human health, and multiple areas have been investigated, with encouraging findings in the suppression of oxidative stress in UC (90). Several studies also demonstrated the significance of probiotics in initiating tolerogenic immune responses and suppressing inflammatory conditions (91). The existence of microbiome composition and species discovered an effective function in attempting to control gut immune response (Figure 3).

### 12.1. Role of synbiotic formulation in the treatment of ulcerative colitis

Few research studies have examined the impact of prebiotic therapy on UC patients so far. The most referenced synbiotic investigations for UC treatments are included in Table 3 (92).

**Table 3:** Summary of essential studies involving synbiotics treatments in ulcerative colitis (UC).

Treatment	Dosage	Duration of treatment	Subjects	Outcomes	References
*Bifidobacterium longum* plus inulin-oligofructose; Treatment time: 1 month	Probiotics: 2 × 10^11^ (CFU) freeze-dried viable *Bifidobacterium longum* and 6 g of prebiotic fructooligosaccharide/inulin mix	1 month/4 weeks	18 patients with active UC	Sigmoidoscopy scores decreased, TNF-α, IL-1βreduced	(92)
*Bifidobacterium longum* plus *psyllium*; Treatment time: 4 weeks	Probiotics: *Bifidobacterium longum*-2 × 10 (9) (CFU), Prebiotics: 8.0-g doses of *psyllium*	4 Weeks	120 patients with UC	IBDQ (total, bowel, systemic, emotional, and social functional scores) increased	(93)
*Lactobacillus Paracasei* B 20160 + XOS; Treatment time: 8 weeks	6 g of lyophilized powder with 5 × 10^9^ CFUs of *Lactobacillus paracasei* B 21060	8 weeks	18 patients with mild-to-moderate UC	Serum IL-6, IL-8 inhibited	(94)
*Bifidobacterium breve* strain Yakult plus galactooligosaccharides; Treatment time: 1 year	1 g of the probiotics powder 10 (9 CFU/g), 5.5 g of GOS	12 months/52 weeks	21 patients with mild to moderate UC	MPO reduction, *Bacteroidaceae* decreased, reduced fecal pH	(95)
*Lactobacillus acidophilus* LA-5^®^, *Lactobacillus delbrueckii* subsp. *bulgaricus* LBY-27, *Bifidobacterium animalis subsp. lactis* BB-12^®^ and *Streptococcus thermophilus* STY-31^™^ plus oligofructose; Treatment time: 1 month	Probiotics: 4 × 10^9^ CFU and pre-biotics 15 g of oligofructose powder	8 weeks	8 patients with UC	Microflora spectrum improved	(96)
*Enterococcus faecium*, *Lactobacillus plantarum*, *Streptococcus thermophilus*, *Bifidobacterium lactis*, *Lactobacillus acidophilus*, *Bifidobacterium longum* plus fructooligosaccharide; Treatment time: 8 weeks	Six probiotic strains: 3 × 10^9^ CFU	8 weeks	40 patients with mild to moderate UC	CRP reduced	(97)
*Streptococcus faecalis* T-110 JPC, *Clostridium butyricum* TO-A, *Bacillus mesentericus* TO-A JPC, *Lactobacillus sporogenes* plus prebiotic; Treatment time: 3 months	The synbiotic capsule contained *Streptococcus faecalis* T-110 JPC: 60 million, *Clostridium butyricum* TO-A: 4 million, *Bacillus mesentricus* TO-A JPC:2 millions, *Lactobacillus sporogenes*: 100 millions	24 Weeks	32 patients with UC	Reduced severity score, steroid intake reduced, relapse during follow-up (3 months) decreased; duration of remission improved.	(98)

## 13. Conclusion

UC is a persistent inflammatory illness with several causes. The principal reason for UC is increased oxidative stress caused by increased ROS production and reduced SOD concentrations. Due to a functioning antioxidant defense mechanism, SOD bioactivity was higher in UC patients. SODs are the primary catalysts that regulate RNS and ROS quantities by directly associating with superoxide and, thus, are essential signaling mediators. As a result, antioxidants may be used in combination with other treatments for UC. Synbiotics function via increasing SOD concentrations, which are primarily accountable for UC.

## Author contributions

SA: writing—original draft, review and editing, and artwork. NM, KKK, ShuR, PS, and PKG: conceptualization, visualization, and supervision. AG, SD, LSW, NA-D, and ShaR: writing—review and editing. BZS, PT, and GR: artwork—figures and editing. All authors contributed to the article and approved the submitted version.

## Conflict of interest

The authors declare that the research was conducted in the absence of any commercial or financial relationships that could be construed as a potential conflict of interest.

## Publisher’s note

All claims expressed in this article are solely those of the authors and do not necessarily represent those of their affiliated organizations, or those of the publisher, the editors and the reviewers. Any product that may be evaluated in this article, or claim that may be made by its manufacturer, is not guaranteed or endorsed by the publisher.

## Abbreviations

UC, ulcerative colitis
NF-κB, nuclear factor kappa-B
IKK, inhibitor of nuclear factor-κB (IκB) kinase
IκB, inhibitor of nuclear factor kappa-B
GIT, gastrointestinal tract
ROS, reactive oxygen species
CAT, catalase
GPX, glutathione peroxidase
SODs, superoxide dismutase
SCFA, short-chain fatty acids
HOCl, hypochlorous acid
H_2_O_2_, hydrogen peroxide
ETC, electron transport chain
COXs, cyclooxygenases
NOS, nitric oxide synthase
MPO, myeloperoxidase
XO, xanthine oxidase
NOX, NADPH oxidase
IECs, intestinal epithelial cells
ICAM, intracellular adhesion molecule
NF-kB, nuclear factor-kappa B
CAMs, complementary and alternative medicines
APC, antigen-presenting cells
TNF-α, tumor necrosis factor-α
IL-1β, interleukin-1β
RCTs, randomized controlled trials
